# Letter from J. W. Craig, Churchville

**Published:** 1857-05

**Authors:** J. W. Craig

**Affiliations:** Churchville


CHURCHVILLE, April 18, 1857.

PROF. S. B. HUNT,

*Dear Sir:* I noticed in your Journal this day received, a communication from Dr. Hamilton, with regard to an epidemic of Cerebro-spinal Meningitis that is occurring in Elmira. For the last three or four weeks it—or something similar to that epidemic—has been in this vicinity. There has been, probably, fifty cases in this and the adjoining towns, death having occurred in from six to ten hours after the attack in five instances. In four of these cases I got a post-mortem examination, and found congestion and softening of the brain in them all; pus formation in one, and effusion of lymph under the pia mater in another. The general symptoms have been a chill, followed by fever, convulsions in children, and delirium in adults, in many cases dilated pupils, strabismus, &c. The pain is very severe in the head, which in a short time extends to the neck, and a sense of soreness and pain is experienced, so that moving the head from the opisthotonotic (I do n't know but I have coined a word) condition, is almost impossible. The pain extends down the back, and rigidity of the muscles soon follow, and in several instances to raise the limbs would be to raise the whole body.

These symptoms give way in from two to ten days, but wandering pains in the bowels and limbs continue for a long time. I have not observed petechiæ in any case, and I have seen the most of the cases that have occurred in this vicinity. But in several instances I have observed an eruption similar to the rose-colored spots (tache rouge) of typhoid fever, which fade in a short time.

The plan of treatment I have adopted has been, in robust subjects, when reäction has fully "come on," to sweat, and give them a mild cathartic, mostly "calomel and oil." This, in many instances, will put a quietus to the difficulty, and if it does not I give them quinine in rather liberal doses. In a few instances I have used stimulants and tonics from the first, but I find that stimulants are not indicated as much as tonics, bnt tonics *are* necessary if they are not relieved during the *congestive stage*, before that of *engorgement*, manifests itself.

I herewith give you a very imperfect description of the disease as it occurs with us, and as the description is to be found in very few books, should you wish, and no one more competent should undertake it, I will furnish you, in my imperfect way, my experience with it.

I hope you will not construe it that I mean any braggadocia, but our individual success had been in forty cases treated but one death, the cases with the one exception had no medicine or died in a few minutes after they were seen by a physician, that proved fatal.

The convalescence, in most of the cases, is very slow.

In haste,

Yours respectfully,

J. W. Craig.

